# Editorial for Special Issue: Microbial and Autoimmune Disease

**DOI:** 10.3390/microorganisms9091800

**Published:** 2021-08-24

**Authors:** Raffaele D’Amelio

**Affiliations:** Department of Clinical and Molecular Medicine, AOU S. Andrea, Sapienza, University of Rome, 00189 Rome, Italy; raffaele.damelio@gmail.com

The relationship between microbial and autoimmune disease is reciprocal and multifaceted, thus it may be interpreted in many ways and developed along different, even opposite, lines (Box 1).

Box 1 **The relationship between microbial and autoimmunity is very tight, bidirectional, as illustrated in the upper part of this figure, and multifaceted, as analyzed in the lower part of the figure and commented in the text.**

**Microbial**

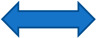

**Autoimmunity**

Infections may induce autoimmunity by different mechanisms including molecular mimicry, bystander activation, epitope spreading [1].Living in dirtier environment seems to be protective towards the appearance of autoimmune diseases (hygiene hypothesis) [2].Autoimmune diseases are at higher risk of infections due to immune dysregulation of the disease itself [3] and immunosuppressive therapy [4].Chronic infections, especially from mycobacteria, may mimic autoimmune diseases [5,6,7].The human microbiota may either, positively, or negatively, according to its composition, modulate the expression of autoimmune diseases [8].Infections may induce a flare-up of a stabilized autoimmune disease, the interpretation of which represents a challenge for clinicians; in fact, it may be due to either the natural history of the disease itself or an infection, two conditions requiring different, rather opposite, therapeutical approaches [9].

In fact, infections have been associated, for a long time, to the induction of autoimmunity, through the demonstrated mechanisms of molecular mimicry and/or bystander activation, and epitope spreading [1]. Conversely, the exposure to a dirtier, thus contaminated by microorganisms, environment has been associated to a protection towards the appearance of allergic and autoimmune diseases, the so-called hygiene hypothesis [2]. A higher risk of infection is generally observed in patients with autoimmune diseases because of the disease itself [3] and immunosuppressive therapy needed to induce the clinical disease remission [4]. Chronic infections, especially from mycobacteria, may mimic systemic autoimmune diseases [5,6,7]. The human microbiota may either, positively, or negatively, modulate the expression of autoimmune diseases [8]. Finally, a frequent challenge for clinicians is the discrimination between spontaneous and infection-induced flares of systemic autoimmune diseases, which imply a different, rather opposite, therapeutical approaches [9].

Thus, in this Special Issue on Microbial and Autoimmune Disease, different aspects of such a multilateral and reciprocal relationship have been considered in five reviews, five original articles and one case report.

So and Tam, from the Chinese University of Hong Kong [10], have provided an updated and detailed review of the relationship between gut microbiome and Spondyloarthritis, an autoimmune inflammatory rheumatic disease occurring with a frequency ranging from 0.2 to 1.6%, mainly in <40-year subjects. The association between Spondyloarthritis and intestinal inflammatory pathologies, such as inflammatory bowel diseases (IBD), has been well-known for a long time. The recent availability of culture-independent molecular methods to investigate the human microbiota, and the best-known gut microbiota, has allowed insight into the role of gut microbiota in modulating the expression of autoimmunity. Different experimental and clinical models are shown, which demonstrate the tight interrelationship between the gut microbiome and the appearance of Spondyloarthritis, including the lack of appearance in germ-free animals. Among the microorganisms, the protective role of *Lactobacillus* is emphasized and among the pathogenetic mechanisms, the role of Paneth and MAIT (mucosal-associated invariant T) cells and of T helper (Th)17, interleukin (IL)17, and IL23, as mediators of damage and local inflammation, is detailed. 

The article by Daniela Melchiorre et al., from Rheumatology of University of Florence [11], explores the role of oral *Lactobacillus* in 29 female patients with Systemic Sclerosis and demonstrates, for the first time, that *Lactobacillus* is significantly reduced compared to 23 age- and sex-matched healthy controls, thus indirectly confirming its probable protective role in a disease model in which oral mucosa may be heavily compromised [12]. However, although *Lactobacillus* has been generally associated with anti-inflammatory activity, and thus protective in the immune-mediated inflammatory diseases, these data have not been consistently confirmed and, precisely in Systemic Sclerosis, gut *Lactobacillus* seems to have an opposite effect, having been found to be increased in contrast with oral *Lactobacillus.*

Meanwhile, in the review of Rosella Mechelli et al. from Neurology of Sapienza University of Rome [13], the role of MAIT cells and microbiota has been investigated in Multiple Sclerosis and other Autoimmune Diseases. In fact, the experimental models used to support the growing role of MAIT cells in Multiple Sclerosis, but even in Systemic Lupus Erythematosus, Rheumatoid Arthritis, Sjögren Syndrome, type 1 Diabetes, and Inflammatory Bowel Diseases are analytically described. Although some studies identify an anti-inflammatory, thus protective, activity by MAIT cells, most studies demonstrate the tight reciprocal relationship between gut microbiota and MAIT cells, which behave as relevant mediators of damage and local inflammation in many autoimmune diseases. 

The influence of the Mediterranean Diet (MD) on the disease activity of 60 patients with Rheumatoid Arthritis (RA) and on their gut microbiota composition was reported by Andrea Picchianti-Diamanti et al. from the Department of Clinical and Molecular Medicine of Sapienza University of Rome [14]. In those RA patients showing a tight adherence to the MD, C-reactive protein (CRP) and Disease Activity Score (DAS) were significantly lower than the values observed in patients who resulted not strictly adherent to MD; even the microbiota was healthier, with a reduction in *Lactobacillus* and *Prevotella copri.* This study confirms previous studies [15,16], which had already found a clinical improvement of RA patients based on the adherence to MD; what is new is the microbiota modification, which represents the mechanism through which MD may exert its favorable effect on RA patients, by reducing inflammation.

A very peculiar aspect of interpreting the relationship between microbial and autoimmune disease is represented by the psychological consequences of the COVID-19-induced lockdown in 100 patients with autoimmune arthritis, compared with 100 controls, analyzed by Andrea Picchianti-Diamanti et al. from the Department of Clinical and Molecular Medicine and Guido Alessandri, from the Department of Psychology of Sapienza University of Rome [17]. Mental health status was measured using the Depression, Anxiety and Stress Scale (DASS-21). The COVID-19 Peritraumatic Distress Index (CPDI) was used to assess the frequency of peritraumatic stress disorders related to COVID-19. Patients reported a significant worsening of perceived General Health (GH) (36% vs. 7%; *p* < 0.001), and a significantly higher mean CPDI score (*p* < 0.001) than controls. Using multivariate analysis, arthritis patients had significantly higher CPDI scores (+3.67 points; *p* = 0.019), independent of depression, anxiety, stress symptoms, comorbidities, sociodemographic, and lifestyle characteristics. Logistic regression analysis showed that the risk of reporting worsened GH was nine-fold higher in patients than controls (*p* < 0.001). Patients with autoimmune arthritis are, therefore, at higher risk of psychological distress related to COVID-19 pandemic; this is a quite unexpected result, considering that patients with autoimmune arthritis have a reduced mobility; thus, it may be believed that they should be slightly affected by the forced inactivity associated with the lockdown, unless such a distress may be principally dependent on the difficulty in maintaining the regularity of the periodical checks, which are needed in chronic diseases. 

The gastric microbiota in the autoimmune gastritis has been reviewed by Conti et al. from the Medical-Surgical Department of Clinical Sciences and Translational Medicine, Sapienza University of Rome [18]. This topic has not been thoroughly explored until recently for several reasons, including the difficulty in culturing bacteria, due to the inhospitable gastric environment for its very low pH. Thus, the issue of gastric microbiota has developed only following the availability of the culture-independent molecular methods. *Helicobacter pylori* remains the most frequently encountered bacterium, but recently even other species of oral origin, such as Streptococci, have been observed, frequently in association with the gastric cancer, so that they have been suspected to be responsible for the cancer induction. Autoimmune gastritis, therefore, with the destruction of parietal cells synthetizing hydrochloric acid, predisposes the stomach to be more easily colonized by bacteria, which may contribute to the cancer development.

We know since long-time that infections may induce, in genetically predisposed individuals, autoimmunity. The first demonstration of the tight link between infections and autoimmunity was the rheumatic fever, for a cross reaction between Group A β-hemolytic *Streptococcus* cell wall/membrane and the cardiac tissue, already observed in 1945, but better defined as molecular mimicry during the second half of the last century, even taking advantage from the availability of immunological tools, such as monoclonal antibodies and T cell clones [19,20]. However, in this model and in most others, the association should first be suspected, then it should be demonstrated by immunological and molecular methods. The case report described in this Special Issue by Biondo et al. from the Department of Clinical and Molecular Medicine, Sapienza University of Rome [21], instead, has benefited from the autoimmune disease being the bullous pemphigoid (BP), an autoimmune cutaneous disease easily identified and diagnosed, and the symptomatic, bacterial infections being quickly and easily diagnosed by culture methods, thus allowing the association to be observed even in real-life conditions. The responsibility of the bacterial infections, especially *Staphylococcus aureus* in blood and *Enterococcus faecalis* in the blister fluid, as BP triggers, may, therefore, be suggested, based on the contemporaneous burst of infections and the autoimmune cutaneous disease. Moreover, even the response to intravenous immunoglobulins (IVIg), which have stabilized the disease within three years from the flare and for 5 years now from the date of any treatment interruption is a further element to support such association. In fact, the disease relapses in 40% of patients within 6 months from the discontinuation of immunosuppressive therapy [22] and treatment with corticosteroids alone has been associated with 40% of mortality per year in elderly patients with BP [23]. The collaboration between dermatologists and immunologists was very fruitful in this case, and the choice of IVIg, at a personalized dose which was half the recommended one to avoid any possible risk of hyper-viscosity in a patient with a history of stroke, was also driven by the multiple infections in a patient with hypogammaglobulinemia. This case report is paradigmatic of the need to always suspect that sudden burst of autoimmune disease may be driven by an underlying infection. A case report of total regression of an early RA following periodontitis treatment only, without any specific therapy for RA [24], is a further witness of the need to always suspect the association, to avoid disease becoming irreversible if the real trigger is not eliminated; rather, the immunosuppressive therapy may make it even more virulent.

However, that IVIg stay at the crossroad of autoimmunity and infections is in their nature of immunomodulating agent, useful as replacement therapy in subjects with hypogammaglobulinemia and infections, and as immunomodulating agent in patients with autoimmunity and it is the guessed title of a well written and updated review by Perricone C et al. from University of Perugia with Triggianese and Perricone R. from the Rheumatology of the University of Rome, Tor Vergata [25]. This review represents an innovative way for interpreting the relationship between microbial and autoimmune disease, it is detailed and offers in one chapter even the landscape on the protective capacity by IVIg, hyper-immune IVIg, hyper-immune plasma from convalescents, and monoclonal antibodies towards COVID-19. Unfortunately, Roberto Perricone, who was full Professor of Rheumatology at the University of Rome, Tor Vergata and a great researcher, but especially a great friend of mine, suddenly passed away on 9 June 2021. He was next to me in different scientific initiatives, including the current Special Issue, with his support and his ideas, thus I would profit of the opportunity of introducing this Special Issue to witness his value as an Immunologist and Rheumatologist, his enthusiasm, his capacity of organizing a highly productive and internationally respected group of research, and his great humanity. 

Ricci et al., from Pneumology of Sapienza, University of Rome [26], through an elegant retrospective study, have observed that nearly a quarter of 44 patients with autoimmune diseases undergoing bronchoscopy for the suspect of interstitial lung disease associated to connective tissue disease were colonized by infectious pathogens, such as *Pseudomonas aeruginosa*, *Hemophilus influenzae* and non-tubercular mycobacteria. These patients had a worse lung function compared with the non-colonized patients, thus suggesting a role of infections in lung disease progression and severity. This study has been carried out in real-life conditions, without the support of culture-independent molecular methods, however the result is of great interest, considering that the literature is quite poor of contributions in this topic.

Canzoni et al., from the Department of Molecular and Clinical Medicine, Sapienza University of Rome [27], analyzed the hepatitis B (HB) serum markers in over 300 patients with different rheumatic diseases, 55% of whom planned for immunosuppressive therapy. Surprisingly, nearly a quarter of them had positivity of HB markers suggestive of a previous contact with HBV, thus underlining the relatively high prevalence of patients at potential risk of reactivation, because of immunosuppressive therapy, despite the availability, since long-time, of an effective HBV vaccine. The vaccination strategy should, therefore, consider immunizing the seronegative adult patients at risk of infection for immunosuppression as well as also the patients with serum traces of a previous contact with HBV resulting non-protective, by trying to further stimulate their exhausted immune system and induce protection in at least some of them. As a serendipitous original observation was the apparent more aggressive clinical course in 9% of patients with relative hypogammaglobulinemia (γ-globulin ≤7 g/dL). 

Finally, Bo et al., from the Department of Biomedical Sciences, Section of Microbiology and Virology, University of Sassari [28], provided an exhaustive and updated review on the role of infections in RA, with a special emphasis on *Mycobacteria*. They remind that the relationship between infections and RA is complex and that infections, frequently linked to immunosuppressive therapy, in addition to the disease itself, may be associated with higher mortality, up to 52%. However, the panoply of microorganisms presented by the authors for which literature exists for a possible etiopathogenetic association with RA is impressive, including *Porphyromonas gingivalis*, *Proteus mirabilis*, *Mycoplasma*, *Bordetella*, *Haemophilus*, *Acinetobacter*, non-tubercular *Mycobacteria*, Parvovirus, Epstein–Barr virus (EBV), Cytomegalovirus (CMV). For non-tubercular *Mycobacteria* and EBV, molecular mimicry has been suggested, with the intervention, in case of non-tubercular *Mycobacteria,* even of bystander activation and epitope spreading.

In conclusion, this Special Issue on Microbial and Autoimmune Disease has attracted valuable scientific contributions, which have faced this topic under different points of view, through interesting and updated reviews, original articles, and one case report in which either traditional or innovative culture-independent technologies have been used, but always able to provide innovative and useful messages, even when deriving observations from the real-life daily clinical activity.
